# Anomalous uptake and circulatory characteristics of the plant-based small RNA MIR2911

**DOI:** 10.1038/srep26834

**Published:** 2016-06-02

**Authors:** Jian Yang, Tremearne Hotz, LaCassidy Broadnax, Mark Yarmarkovich, Ismail Elbaz-Younes, Kendal D. Hirschi

**Affiliations:** 1 USDA/ARS Children’s Nutrition Research Center, Baylor College of Medicine, 1100 Bates Street, Houston, TX 77030, USA; 2 Bates College, 2 Andrews Rd, Lewiston, ME 04240, USA; 3 Cancer Biology, University of Pennsylvania, 3501 Civic Center Boulevard, Philadelphia, PA 19104, USA; 4 Vegetable and Fruit Improvement Center, Texas A&M University, College Station, TX 77845, USA.

## Abstract

Inconsistent detection of plant-based dietary small RNAs in circulation has thwarted the use of dietary RNA therapeutics. Here we demonstrate mice consuming diets rich in vegetables displayed enhanced serum levels of the plant specific small RNA MIR2911. Differential centrifugation, size-exclusion chromatography, and proteinase K treatment of plant extracts suggest this RNA resides within a proteinase K-sensitive complex. Plant derived MIR2911 was more bioavailable than the synthetic RNA. Furthermore, MIR2911 exhibited unusual digestive stability compared with other synthetic plant microRNAs. The characteristics of circulating MIR2911 were also unusual as it was not associated with exosomes and fractionated as a soluble complex that was insensitive to proteinase K treatment, consistent with MIR2911 being stabilized by modifications conferred by the host. These results indicate that intrinsic stability and plant-based modifications orchestrate consumer uptake of this anomalous plant based small RNA and invite revisiting plant-based microRNA therapeutic approaches.

Evidence has been presented that small RNAs ingested from plant-based foods act as bioactives^1^,^2^,^3^,^4^. Concurrently, numerous groups have failed to detect any dietary RNAs in plasma^5^,^6^,^7^. The most compelling evidence regarding activity of *exogenous* plant based small RNAs called *microRNAs (miRNAs)* from plant-based foods is work done with rice-based miRNA MIR168a and the herb-based 26S ribosomal RNA-derived small RNA, termed miRNA MIR2911^1^,^2^. One group has reported that both MIR168a and MIR2911 are absorbed by cells of the gastrointestinal (GI) tract of consuming animals, packaged into exosomes, and trafficked via the bloodstream to a variety of tissues, where they are capable of regulating mammalian genes^1^,^2^.

While, numerous groups have failed to detect any dietary RNAs in plasma^5^,^6^,^7^, independent studies have specifically replicated the detection of circulating plant-derived MIR2911 which is found in high levels in various herbs^8^,^9^,^10^. The intensity of detection of MIR2911 in murine sera and urine are approximately correlated with dietary intake^9^. However, rote abundance is not the sole determinate of apparent RNA uptake, as gavage-feeding large doses of synthetic MIR2911 permitted only small transient increases in serum levels^9^. Moreover, the fact that MIR2911 is one of the few dietary plant RNAs whose uptake by animal consumers has been replicated poses the question of whether the efficacy of MIR2911 delivery to animals might be attributed to unique characteristics of this small RNA. Specifically, unique plant derived packaging and/or the intrinsic stability conferred by the nucleotide sequence and composition of MIR2911 could contribute to its enhanced uptake compared to other miRNAs.

RNAs in circulation are considered to be unstable molecules that are subject to prompt degradation by endogenous RNases and rapid renal clearance^11^. However, intact RNAs of various types have been detected in circulation^12^. Characterization of the extracellular miRNAs in peripheral blood plasma suggests nuclease resistant miRNAs exist in different forms, such as in exosomes or in association with components of the RNA-induced silencing complex (RISC), *e.g.* Argonaute 2 (AGO2)^13^,^14^. However, in disagreement with others, our previous work shows that circulating dietary MIR2911 does not co-immunoprecipitate with AGO2^1^,^9^. Further characterization of circulatory traits of MIR2911 is critical to our understanding of the mechanisms of dietary plant-based small RNA uptake.

We report here an investigation of the physical state of MIR2911 in both plants and in circulation. We measured digestive stability and uptake of the plant-derived MIR2911 in comparison to synthetic MIR2911 and other plant miRNA species. Together the results of the present study demonstrate dietary MIR2911 is packaged and bioavailable from numerous widely consumed vegetables. Furthermore, the MIR2911 complex maybe modified by the host to facilitate circulatory stability. Our results highlight unique plant-based modifications, RNA stability, and host specific alterations as possible prerequisites for detection of a circulating plant-based small RNA and offer a plausible explanation for the inability of numerous groups to detect dietary miRNAs in animal sera.

## Results

### Assaying 2911 levels in vegetables

Various herbs that are not widely consumed in the western diet have been shown to contain high levels of MIR2911^9^. Here, different vegetables, purchased from a local grocery store, were assayed for levels of MIR2911 via quantitative reverse transcription-polymerase chain reaction (qRT-PCR) (Table 1). Cauliflower displayed the highest levels of MIR2911 at 549 fmoles/g while broccoli had 302 fmoles/g, cabbage had 380 (fmoles/g), spinach had 228 fmoles/g and lettuce had 84 fmoles/g of MIR2911.

### Uptake of plant-based mir2911 in animals fed vegetable diets

**Figure 1:**
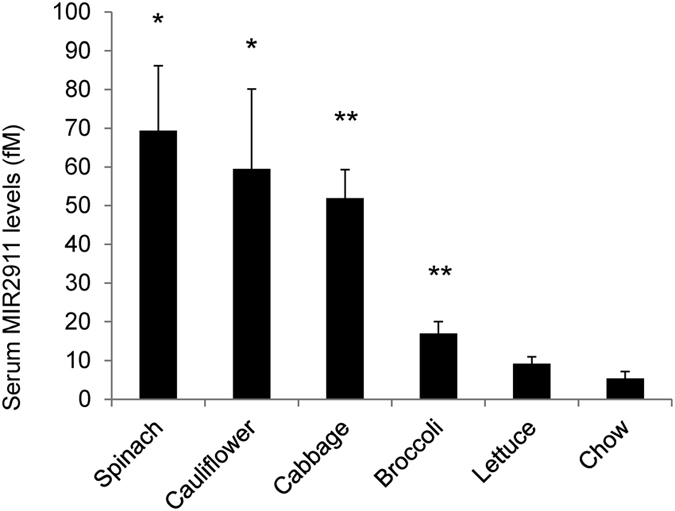
Serum MIR2911 in mice fed various vegetable-containing diets. Detection of MIR2911 in sera from mice fed various vegetable-containing diets. Mice were fed diets for 7 days before blood collection and qRT-PCR analysis. N = 5; error bars = S.E.M. *p < 0.05; **p < 0.01 between chow and vegetable diet fed mice. Experiment replicated at least three times with each diet.

Using a modified chow-based diet supplemented with vegetables, we tested whether MIR2911 could be detected from sera of animals consuming these diets. MIR2911 was measured seven days after initiating feeding. Fold differences in circulating MIR2911 levels in vegetable diet-fed animals compared to chow-fed animals were as follows: spinach, 13-fold higher (69 femtomolar (fM)); cauliflower, 11-fold higher (60 fM); cabbage, 10-fold higher (52 fM); broccoli, 3-fold higher (17 fM). A small change was detected in lettuce fed mice (9 fM) (Fig. 1).

### Content of MIR2911 in edible plant derived exosome-like nanoparticles (EPDENs) and the form of MIR2911 in cabbage extracts

**Figure 2:**
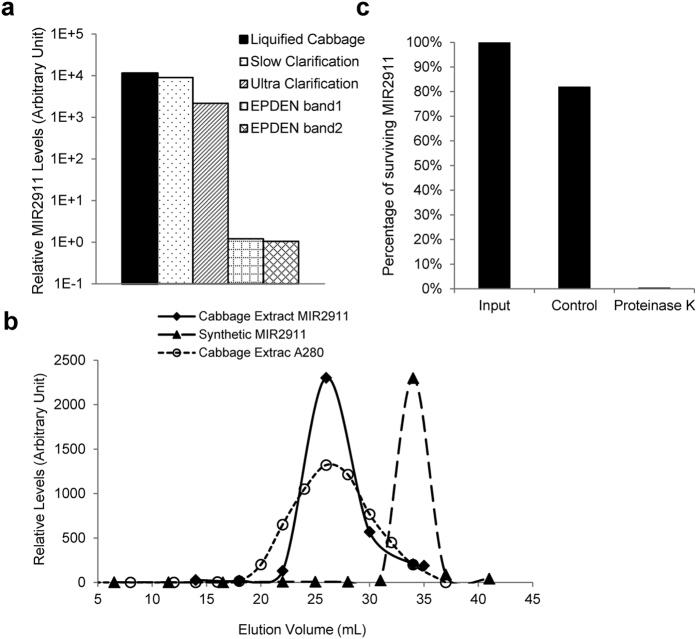
Forms of MIR2911 in cabbage extract. (**a**) Edible Plant Derived Exosome-like Nanoparticles (EPDENs) were purified from clarified cabbage lysate via ultracentrifugation. Levels of MIR2911 were quantified by qRT-PCR in various fractions: cabbage leaves liquefied in a blender (Liquefied Cabbage); Supernatant from 10,000 × g centrifugation (Slow Clarification); Supernatant from 150,000 × g ultracentrifugation (Ultra Clarification); EPDEN bands at 30% 45% interface (EPDEN band1) and 45% 60% interface (EPDEN band2) of sucrose gradient fractionation of total vesicles. (**b**) Size exclusion chromatography analysis of MIR2911 in ultracentrifugation-clarified cabbage lysate (**c**) qRT-PCR analysis of MIR2911 levels in proteinase K-treated or control (PBS-treated) ultracentrifugation-clarified cabbage lysate.

Previous work has shown that edible plant derived exosome-like nanoparticles (EPDENs) contain miRNAs and inferred that they may serve as a vehicle to deliver plant dietary miRNAs to animals^15^,^16^. We sought to determine if MIR2911 within plants was encapsulated in these exosome-like particles. Using standard ultracentrifugation-based exosome purification techniques^16^, we isolated EPDENs from cabbage extract. The particles were characterized as exosome-like based on electron microscopic examination of sucrose gradient purified bands (Supplementary Fig. 1a). However, the majority of MIR2911 was not detected in the EPDENs but found mainly in the non-vesicular fractions (Fig. 2a). As an alternative approach, size exclusion chromatography (SEC) was applied to the cabbage extract (Fig. 2b) to discern the complexation of MIR2911 in the plant. Comparing the MIR2911 elution profile with those of the size standards and free synthetic RNA (Supplementary Fig. 2, Fig. 2b), the data suggested that the size of MIR2911 found in cabbage extracts is consistent with that of a protein-containing complex. Indeed, MIR2911 from the cabbage extract appeared to be sensitive to proteinase K treatment that reduced MIR2911 content by more than 99%, while only 18% of the MIR2911 was degraded in the control (Fig. 2c). These studies demonstrated that MIR2911 is not contained within EPDENs in plants, but is complexed, possibly with a protein.

### Assaying stability of MIR2911 using *in vitro* digestion

**Figure 3:**
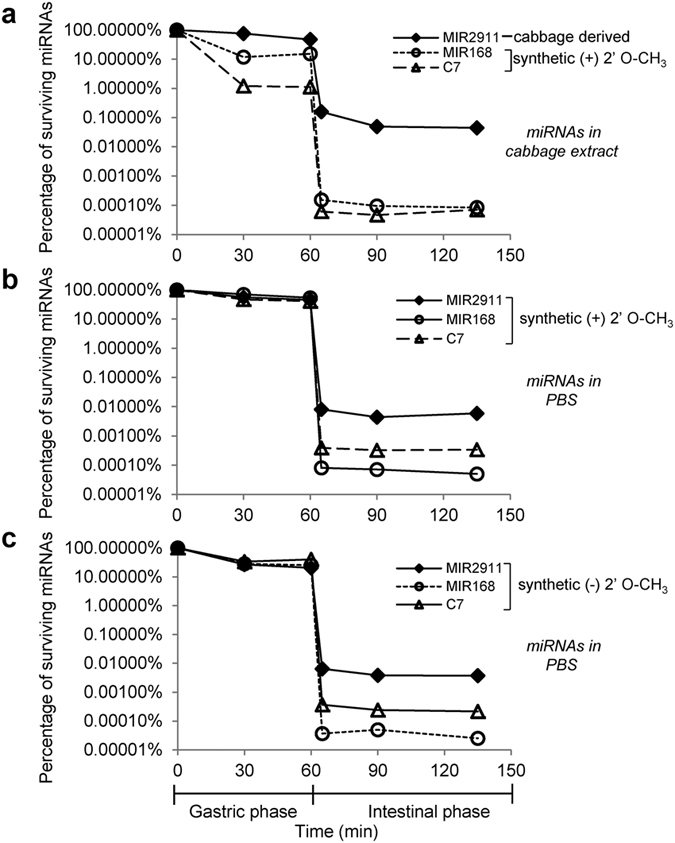
Superior Stability of MIR2911 compared to other plant microRNAs in *in vitro* digestion. The stability of MIR2911, MIR168 and C7 were assayed in an *in vitro* digestion system that includes a gastric digestion phase and a intestinal digestion phase. Three different samples were digested: (**a**) cabbage lysate containing a measured amount of 10 pmoles of plant-derived MIR2911 and spiked-in 10 pmoles each of synthetic 2′ O-methylated MIR168 and C7; (**b**) 10 pmoles each of synthetic 2′ O-methylated MIR2911, MIR168 and C7; (**c**) 10 pmoles each of synthetic MIR2911, MIR168 and C7 without 2′ O-methylations. Aliquots of digestion reaction were removed at various time points for analysis: 30 min, 60 min in gastric phase, 5 min, 30 min, and 75 min in intestinal phase. Levels of surviving miRNAs were measured by qRT-PCR. The surviving percentages were calculated by comparing the levels of miRNAs at each time point to their starting levels before digestion.

The plant-based modifications of MIR2911 may render it more resistant to digestion and contribute to the enhanced serum detection reported previously^8^,^9^. It also remains uncertain whether post-translational miRNA modifications (such as 2′ O-methylation at the 3′ nucleotide) protect the plant-based small RNA from degradation, and whether the sequence or nucleotide composition of MIR2911 confers stability since compared with other miRNAs, MIR2911 appeared to be more robustly absorbed than other plant miRNAs^8^,^9^. To address these questions, an artificial *in vitro* digestion system that simulates mammalian gastric and intestinal conditions was employed^17^. Three samples were compared: sample one contained 1 mL of cabbage extract that contained 10 pmoles of plant derived MIR2911 as measured by qRT-PCR, and 10 pmoles of spiked-in synthetic 2′ O-methylated MIR168a and artificial miRNA termed C7; sample two contained 1 mL of phosphate buffered saline (PBS) with 10 pmoles each of synthetic 2′ O-methylated MIR2911, MIR168a and C7; sample three contained 1 mL of PBS with 10 pmoles each of synthetic MIR2911, MIR168a and C7 without the 2′ O-methylation. Time course analysis of the miRNA content was done during both gastric and intestinal phases of digestion. Most miRNAs displayed modest resistance in the acidic gastric environment, while in the intestinal phase the levels of all miRNAs were drastically reduced after five minutes. However, regardless of synthesis origin and the 2′ O-methylation, the digestive stability of MIR2911 was 10–100 fold higher than that of the other miRNAs tested. For MIR2911, the most stable form was the plant derived form in the cabbage extract, as 0.044% survived, compared to 0.0059% for the 2′ O-methylated form, and 0.0037% for the non-modified form (Fig. 3).

### MIR2911 stability *ex vivo* and *in vivo*

**Figure 4:**
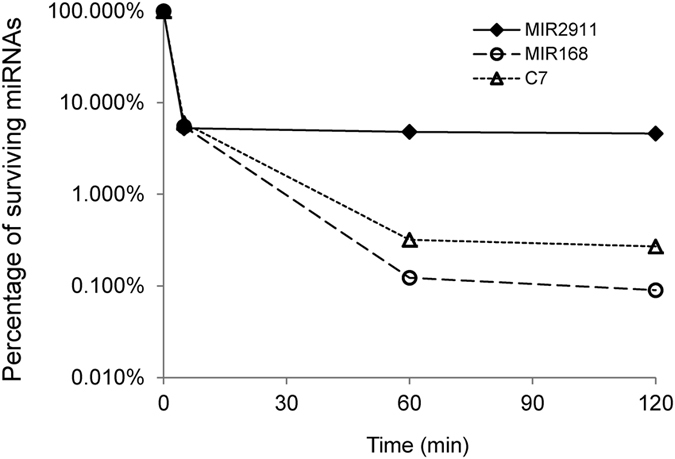
Stability of miRNAs assayed in *ex vivo* intestinal fluids. 10 pmoles of synthetic 2′ O-methylated MIR2911, MIR168a and C7 were incubated in mouse *ex vivo* intestinal fluids. Surviving miRNA levels were assayed at 5 min, 60 min and 120 min during digestion; percentage of survival were calculated by dividing surviving levels to the measured pre-digestion levels.

MIR2911 displayed greater stability during *in vitro* digestion (Fig. 3), we thus sought to test whether MIR2911 was more stable than other miRNAs in the murine gut. 10 pmoles of synthetic 2′ O-methylated MIR2911, MIR168a and C7 were digested in the *ex vivo* intestinal fluids and were tested for surviving miRNA levels. The results from the *ex vivo* digestion agreed with the *in vitro* digestion in that MIR2911 appeared more stable after 2 hours of digestion than the other miRNAs tested (Fig. 4).

**Figure 5:**
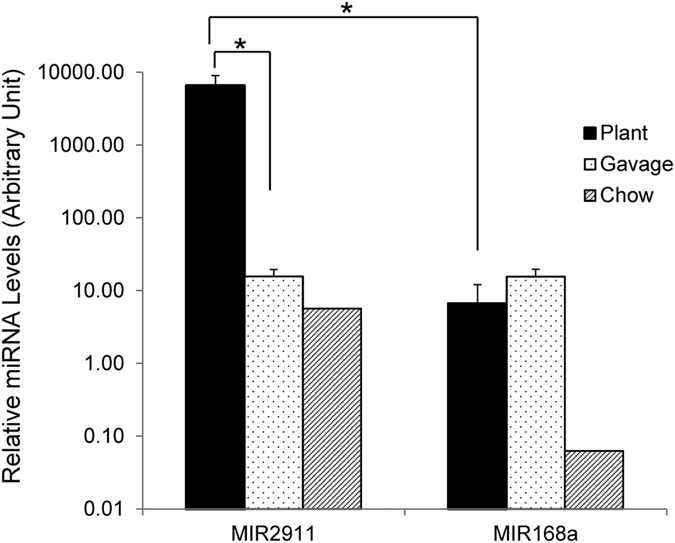
Mice fed plant diets contain substantially higher MIR2911 in the small intestines than MIR168a, and than mice gavage fed a single dose of microRNAs. Levels of MIR2911 and MIR168 in the small intestines were measured by qRT-PCR from mice fed honeysuckle diet (Plant), mice gavage fed synthetic miRNAs (Gavage), or mice fed the control chow diet (Chow). N = 3; error bars = S.E.M. *p < 0.05 between Plant and Gavage, or Plant and Chow groups.

A greater efficiency for MIR2911 uptake was previously observed through feeding plant based diets than through gavage feeding of synthetic RNAs^9^. This observation prompted a further comparison between the stability of MIR2911 from a plant-based diet and synthetic MIR2911 gavage fed to the mice, by directly measuring MIR2911 levels in the small intestines of mice under these feeding conditions. It was found MIR2911 levels in the small intestines of mice fed the plant-based diet (daily dietary MIR2911 intake 10 pmoles) were more than a 100-fold higher than those of mice gavage fed a single 400 pmole dose of the synthetic MIR2911 (Fig. 5). MIR168a, a miRNA present in both plant-based diets and chow, was detected in the small intestines at a level 100-fold lower than that of MIR2911 (Fig. 5). Together, these results substantiate that MIR2911, especially MIR2911 derived from plants, is more stable than other plant-based miRNAs.

### Uptake of plant-derived MIR2911 vs synthetic MIR2911

**Figure 6:**
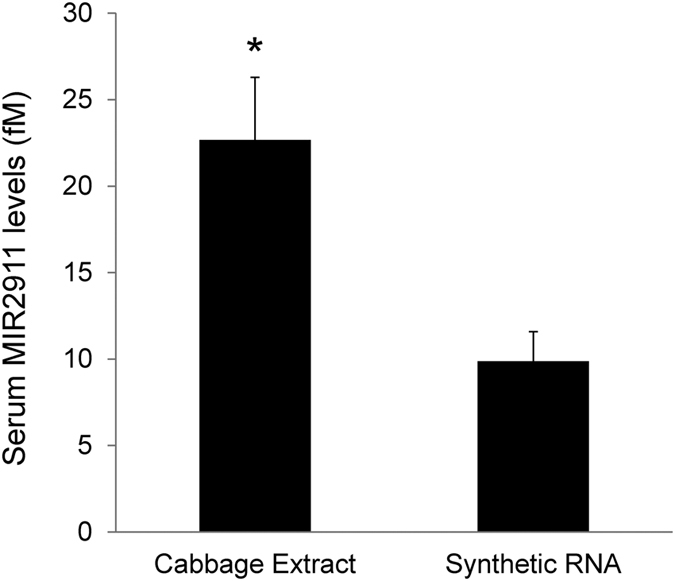
Enhanced detection of MIR2911 in mice gavage fed concentrated cabbage extract compared to synthetic RNA. 12.5 pmoles of MIR2911 were contained in each concentrated cabbage extract dosage, while 400 pmoles of MIR2911 were contained in each synthetic RNA dosage. N = 5; error bars = S.E.M. *p < 0.05.

Stability of plant-derived MIR2911 in cabbage extracts was greater than that of the synthetic MIR2911 (Fig. 3); however does this translate into improved bioavailability? To test this, we sought to compare and contrast uptake efficiency of MIR2911 in these two forms by gavage feeding. To achieve a higher feeding dose, cabbage extracts were concentrated to contain 12.5 pmoles of MIR2911 per 500 μL gavage volumes. As a comparison, similar quantities of synthetic MIR2911 should be used; however, this dose has proven to be insufficient to render detectable MIR2911 levels in the sera of animals^9^. Hence we chose higher dosages of synthetic MIR2911 (400 pmoles per mouse, 32-fold more than the cabbage MIR2911 dosage). An approximate 2-fold increase in the serum MIR2911 levels from the mice gavage fed cabbage extract was detected compared to the animals receiving synthetic 2′ O-methylated MIR2911 (Fig. 6). This suggested that MIR2911 derived from plants is more bioavailable than the synthetic RNA.

### Characterization of circulating MIR2911

**Figure 7:**
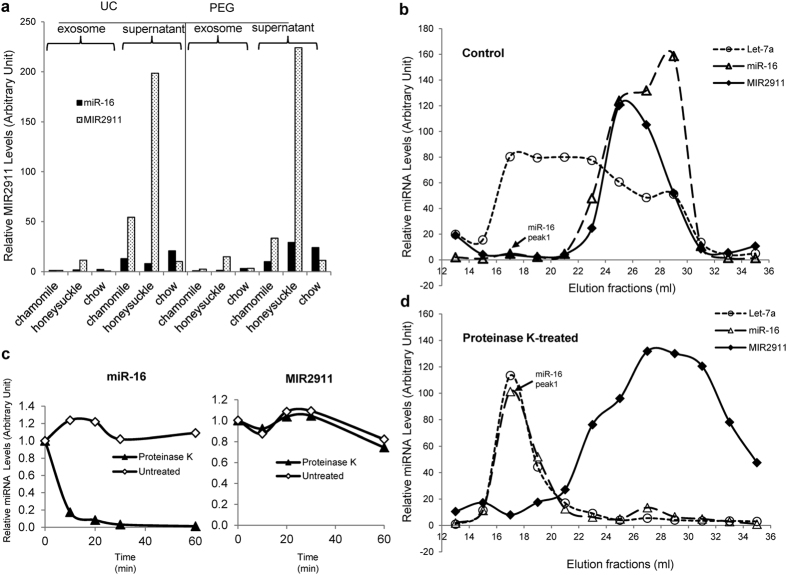
Circulatory forms of MIR2911 in mice fed plant-containing diets. (**a**) Sera were obtained from mice fed either chamomile, honeysuckle, or chow diet. Exosomes were isolated from these mouse sera via ultracentrifugation protocol (UC) or PEG precipitation protocol (PEG). Levels of miR-16 and MIR2911 were quantified by qRT-PCR in the exosome pellets, or the supernatant fractions. (**b**) Size exclusion chromatography analysis of Let-7a, miR-16 and MIR2911 levels in control (untreated) serum samples from mice fed vegetable diets. (**c**) Time course analysis of miR-16 and MIR2911 levels in proteinase K-treated or control (untreated) serum samples from mice fed vegetable diets. (**d**) Size exclusion chromatography analysis of Let-7a, miR-16 and MIR2911 levels in proteinase K-treated serum samples from mice fed vegetable diets. Note: in Panel (**b**) and (**d**), the scale for Y axis for the 3 miRNA targets are set independently. E.g. for miR-16, the peak1 in panel (**b**) (arrow) represents similar concentration as the peak1 in panel (**d**) (arrow).

We initially hypothesized that dietary MIR2911 is present within circulating Argonaute complexes. However, in earlier work we failed to co-immunoprecipitate MIR2911 with antibodies against a mouse AGO2 protein^9^. Here we tested whether circulating MIR2911 is primarily encapsulated in exosomes or stabilized by other proteins. We used both differential ultracentrifugation and PEG precipitation-based methods to purify circulating vesicles from mice fed cabbage and various herbs. The recovery of vesicles consistent in size and morphology with exosomes and microvesicles was confirmed by transmission electron microscopy (TEM) (Supplementary Fig. 1b) and an exosome quantification kit (Supplementary Fig. 1c). Vesicle pellets and the vesicle-poor supernatants were assayed via qRT-PCR for miR-16, a well characterized circulating endogenous miRNA^14^. In the context of either of the two exosome isolation protocols, the majority (over 90%) of the circulating miR-16 and MIR2911 were not found in the exosome vesicles (Fig. 7a). To further understand the circulatory forms of MIR2911, size exclusion chromatography (SEC) was employed. Given the large size of exosome associated miRNAs, they should elute from SEC columns early relative to protein associated miRNAs such as miR-16. Using pooled MIR2911-rich mouse sera, miRNA-containing particles were fractionated. The copies of MIR2911, miR-16, and let-7a (an exosome-associated miRNA), were quantified in each fraction by qRT-PCR. The results showed that let-7a eluted earlier than both MIR2911 and miR-16. The fractionation of let-7 and miR-16 were consistent with previous work in human plasma^14^ while MIR2911 fractionated in the vicinity of the protein associated miRNA miR-16 (Fig. 7b).

### A protease-insensitive complex stabilizes circulating MIR2911

We sought to further address the putative protein association of MIR2911 in sera by proteolytic digestion. If a protein complex protects MIR2911 then proteinase K treatment would render MIR2911, like miR-16, sensitive to degradation by plasma RNases^14^. In our assay conditions miR-16, previously shown to bind to AGO2, was rapidly degraded in the presence of proteinase K; however, MIR2911 was insensitive to this treatment (Fig. 7c). When the proteinase K-treated sera were run on the SEC column, the non-exosome elution peaks of miR-16 and Let-7a disappeared compared to the control sera; however, the eluting pattern of MIR2911 remained largely unchanged (Fig. 7b,d). This alteration in the physical state of MIR2911, transitioning from a proteinase K-sensitive complex in cabbage extracts (Fig. 2c) to a proteinase K-resistant complex in circulation implies a host modification of the dietary small RNA during uptake.

## Discussion

Exposure to dietary MIR2911 can come from a variety of popular vegetables (Table 1). This plant-derived small RNA delivered by typical dietary ingestion had a digestive robustness that appears to help make it more bioavailable compared to other miRNAs (Figs 3 and 4). In milk, specific miRNAs appear to be encapsulated in exosomes conferring protection against degradation and facilitating uptake^18^,^19^. We hypothesize that a plant specific protein can facilitate uptake as well as enhancing stability during digestion. Our results implicate modification of the MIR2911 complex during consumer uptake. During digestion or absorption, the proteinase K-sensitive cabbage complex (Fig. 2) is transformed into a serum-stable proteinase K-resistant complex (Fig. 6). We speculate this protein “shield” of MIR2911may be modified in transit, possibly within intestinal epithelial cells. It remains an open question whether the plant binding protein is completely exchanged with an animal protein, or it is only chemically modified.

Different plant-based miRNAs are present within the cellular matrix of the plant material and may be associated with other plant molecules such as proteins and polysaccharides^20^. These components along with post-translational modifications may provide a conduit for dietary plant-derived miRNAs uptake^20^. In previous *in vitro* experiments, miR168a in soybean and mir166 in rice display a greater resistance to degradation than miR168a from rice and mir166 from soybean^20^. Given that there are no sequence differences between these soybean and rice miRNAs it has been proposed that plant-specific mechanisms are in play that offer varying protection of miRNAs from degradation. Here we also demonstrate the importance of the primary sequence, as MIR2911 is approximately 100-fold more tolerant to simulated mammalian intestinal digestion conditions than MIR168a (Fig. 3). This tolerance is independent of the 2′ O-methylation as the synthetic non-methylated form is equally stable (Fig. 3).

Endogenous extracellular miRNAs are often associated with ribonucleoprotein complexes or exosomes in the circulation as this packaging protects the small RNA from RNases^14^. We discovered here that the majority of plant-derived MIR2911 appeared to circulate independently from either of these mechanisms. Using SEC fractionation of sera from mice on vegetable diets, MIR2911 did not migrate with the exosome-associated miRNA let-7a (Fig. 6b,d). Meanwhile, although MIR2911 did fractionate with miR-16, an AGO2 associated miRNA, MIR2911 was not proteinase K sensitive (Fig. 6c,d). Together these results suggested the majority of MIR2911 in circulation was in a unique form distinct from these endogenous circulating miRNAs. Additional studies will be needed to unravel mechanisms in the GI tract that facilitate this dietary small RNA uptake and identify how this small RNA is stabilized by the host to facilitate its circulatory stability. Previously, we have sequenced MIR2911 from the sera to show that consumer uptake does not alter the sequence of the small RNA^9^. However, our methodologies are only measuring levels of full length MIR2911 and truncations and mutant versions of MIR2911 may also be bioavailable.

The unique properties of MIR2911, especially the superior stability against digestion, are not universal to all plant-based miRNAs (Figs 3 and 4) and thus might explain the discordance in detection frequencies among dietary plant-based miRNAs in circulation^21^. This could explain why serum detection of plant miRNAs has proven problematic^5^,^6^,^7^. Edible plant derived exosome-like nanoparticles (EPDENs) appear to mediate interspecies communication^16^ but it remains an open question if these effects are mediated by small RNAs. In our case, the majority of MIR2911 did not appear to be physically associated with EPDENs (Fig. 2).

Considering the superior uptake efficiency of the anomalous MIR2911, it can serve as a model for reverse engineering of therapeutic small RNAs that are stable in peripheral blood. Future work can focus on investigating mechanisms of digestive stability and uptake, and testing the functionality of dietary plant small RNAs in consumers.

## Methods

### Animal studies

All studies involving mice were carried out in accordance to the approved guidelines of the Institutional Animal Care and Use Committee (**IACUC**) of Baylor College of Medicine. The experimental protocol involving mice was approved by **IACUC** of Baylor College of Medicine. Specifically, the institutional animal protocols AN-2624, AN-6438 and AN-6454 cover the experiments performed in this study. Male 7- to 8-week-old ICR mice were used and were obtained from the Center for Comparative Medicine at Baylor College of Medicine. All feeding studies were replicated at least three times and the results shown are representative of the biological replicates. Vegetables used in the feeding studies were obtained from a local grocery store, and were freeze-dried to 30% of the fresh weight before they were finely ground and used to prepare diets. The plant-chow diets were prepared by mixing finely ground chow, plant material, and water at 2:1:2 weight ratios^9^. Each day 5 g of the plant-chow diet that contained 1 g of dried plant material was fed to each mice. The daily intake of plant material per each mouse is equivalent to 3.3 g of fresh weight.

### Serum collection and RNA extraction

Blood was collected via retro-orbital bleeding of mice and was allowed to coagulate at room temperature for 1 h^9^. Sera were separated by centrifugation at 800 × *g* for 10 min at room temperature followed by centrifugation at 10,000 × *g* at 4 **°**C for 10 min to remove all blood cells and debris. Total RNA was extracted from 100 μL of sera using the miRNeasy Mini Kit from Qiagen (Valencia, CA) following manufacturer’s recommendations. Exogenous control RNAs were spiked in at 1 pmole per sample after addition of Qiazol to the samples.

### Analysis of miRNA levels by qRT-PCR

Taqman microRNA Assays for let-7d,g,i^22^, let-7a, miR-16, MIR161, MIR2911, MIR168a, and C7 were obtained from Life Technologies (Grand Island, NY). For serum samples, total RNA equivalent to 10 μL of sera was used in each RT reaction. Of the 10 μL RT product, 1 μL was used for each triplicated qPCR reaction. To quantify miRNA levels in the plants, 10–30 mg of fresh plant material were ground to a fine powder in liquid nitrogen and then subjected to RNA isolation using the miRNEASY kit (Qiagen, Valencia, CA); 1 pmole of synthetic artificial miRNA C7 was spiked into the plant Qiazol lysate as an exogenous RNA control. The quantification result was normalized to the weight of starting plant material. qRT-PCR was performed using a Biorad CFX96 Real-Time PCR Detection System, and data were analyzed using Biorad CFX software^9^. The Delta-Delta-Ct method was used to calculate relative levels of miRNAs. Absolute concentrations of miRNAs were calculated based on standard curves obtained from serial dilutions of synthetic miRNAs. The quantification Ct values all fall in the linear range of taqman assays as determined by the synthetic microRNA standards.

### Preparation of synthetic miRNAs

Synthetic miRNAs were obtained from Integrated DNA Technologies (Coralville, IA). The sequence of the miRNAs are as follows: MIR-2911 5′-GGC CGG GGG ACG GGC UGG GA-3′; MIR-168a 5′-UCG CUU GGU GCA GAU CGG GAC-3′; C7 5′-GGA UCA UCU CAA GUC UUA CGU3′; MIR161 5′- UCA AUG CAU UGA AAG UGA CUA-3′. For gavage feeding, the RNAs were diluted in RNase-free PBS, and each animal was fed 400 pmoles of each RNA in 500 μL volume.

### Preparation and concentration of cabbage extract, and isolation of Edible Plant Derived Exosome-like Nanoparticles (EPDENs)

Fresh cabbage was purchased from a local market, mixed with ice-cold water at 1 g/mL, and then blended in a mixer. This slush was sequentially centrifuged at 1000 × g for 10 min, 3000 × g for 20 min, and 10,000 × g for 20 min to remove large particles. The supernatant was then centrifuged at 150,000 × g for 90 min. Supernatant from the ultracentrifuged sample was denoted cabbage extract. MIR2911 was concentrated from 50 mL of cabbage extract to 5 mL using an EMD Millipore (Billerica, MA) Amicon protein concentrator with a molecular weight cutoff of 30 kD following the manufacturer’s specifications. EPDENs were isolated and purified from the pellet from the ultracentrifugation according to the methods as described previously^16^. The pellet was resuspended in PBS, transferred to a sucrose step gradient (15%/30%/45%/60%) and centrifuged at 150,000 × g for 120 min using a previously described protocol^16^. The bands between the 8%/30% layer and 30%/45% layer were harvested separately and noted as EPDENs. The protein concentration of these fractions was determined using the Bio-Rad protein quantification assay kit (Bio-Rad Laboratories, Hercules, CA, USA). The purified specimens were prepared for electron microscopy using conventional procedures^14^ and observed using an Hitachi H7500 Transmission Electron Microscope. Photomicrographs were taken using a AMT XR-16 digital camera.

### *In vitro* digestion of miRNAs

*In vitro* digestion conditions were based on a previously described protocol^14^,^14^. Briefly, the gastric phase was composed of a gastric electrolyte solution (7.8 mM K^+^, 72.2 mM Na^+^, 70.2 mM Cl^-^, 0.9 mM H_2_PO_4_^-^, 25.5 mM HCO_3_^-^, 0.1 mM Mg^2^^+^, 1.0 mM NH_4_^+^, 0.15 mM Ca^2^^+^) with pH adjusted by 1N HCl to 2.0, and porcine pepsin (80 mg/mL) (Sigma, St. Louis, MO); the intestinal phase was formed by adding to the gastric phase an intestinal electrolyte solution (7.8 mM K^+^, 72.2 mM Na^+^, 124.4 mM Cl^-^, 55.5 H_2_PO_4_^-^, 85 mM HCO_3_^-^, 0.33 mM Mg^2^^+^, 0.6 mM Ca^2^^+^), 24 mg/mL of bile extract (Sigma, St. Louis, MO) and 4 mg/mL of porcine pancreatin (Sigma, St. Louis, MO), and 1N NaOH to adjust the pH to 7.0. 1 mL of cabbage extract spiked in with synthetic miRNAs or synthetic miRNAs in a 1 mL PBS solution were first mixed with 1 mL gastric phase and digested with slow rotation at 37 °C for 60 min. The digestion mixture was then mixed with the intestinal phase and digested with slow rotation at 37 °C for an additional 75 min. 100 μL of samples were removed at 30 min, 60 min of the gastric phase, and 5 min, 30 min and 75 min of the intestinal phase for analysis of surviving miRNA levels. 100 μL of pre-digestion samples were used as controls for calculating the percentage of surviving miRNAs.

### Proteinase K treatment

Mouse sera or cabbage extract were mixed with proteinase K (P1807S, New England Biolabs, Ipswich, MA) at a final concentration of 1 mg/mL, or with the control PBS buffer and were incubated at 55 °C for 1 hour.

### Digestion of miRNAs in *ex vivo* intestinal fluids; assaying miRNA levels in the small intestines *in vivo*

Intestines from chow-fed mice were excised and flushed with 1 mL PBS to collect the contents of the intestines. The supernatant derived from centrifugation at 10,000 × g for 5 min was denoted *ex vivo* intestinal fluid. 10 pmoles of synthetic 2 O’-methylated MIR2911, MIR168a and C7 were incubated in mouse *ex vivo* intestinal fluids with slow rotation at 37 °C. Residual intact miRNA levels were assayed at 5 min, 60 min and 120 min of digestion; percentage of residual intact miRNA was calculated by dividing surviving levels to the measured pre-digestion levels. For assaying miRNAs from small intestines, mice were fed a honeysuckle diet or chow diet for 3 days^8^. The mice were then sacrificed and intestinal contents collected and miRNA levels measured. Simultaneously, chow-fed mice were gavaged 400 pmoles of MIR2911 and MIR168a. miRNA levels from the intestine were assessed 2 hours post gavage.

### Isolation of serum exosomes

Exosomes were isolated by two methods: ultracentrifugation^14^ and PEG precipitation (Serum Total Exosome Isolation Kit (Invitrogen, Carlsbad, CA)^23^. Briefly, for the ultracentrifugation method, 1 mL of pooled sera was diluted by DEPC-treated sterile PBS to 3 mL and was then clarified at 12,000 × g for 15 min. The supernatant was then centrifuged at 190,000 × g for 2 h. The vesicle pellet was resuspended in 400 μL PBS. For the PEG precipitation, 200 μL of pooled serum was clarified at 2000 × *g* for 30 min after which the exosomes were isolated according to the manufacturer’s instructions. As quality controls, exosome vesicles were fixed, contrasted with uranyl oxalate, and visualized by transmission electron microscopy. Additionally, the exosome preparation were quality-checked and quantified by a commercially available EXOCET kit (SystemBio, Mountain View, CA) following manufacturer’s instructions.

### Size-exclusion chromatography

A Sephacryl S-500 column (GE Healthcare, Pittsburgh, PA) was loaded with 1 mL of pooled mouse serum, or cabbage extract and eluted with PBS solution (pH 7.4) at room temperature^14^. To verify the efficacy of the SEC column, the elution pattern of size standards including thyroglobulin (660 kD), BSA (66.5 kD) and tyrosine (0.181 kD), and a mouse serum sample were analyzed. The results agreed with a previous report, demonstrating that the SEC column is adequate to separate vesicle and protein fractions^14^. The copies of MIR2911, miR-16, and let-7a were quantified in each fraction by qRT-PCR and normalized to spiked-in 161 miRNAs. Protein abundance of each fraction was measured by absorbance at 280 nm. Fractions were stored at −20 °C before use.

### Statistical analysis

Statistical analyses were performed with the student T-Test formula in Microsoft Excel. Significance was set at P < 0.05. Data are presented as means ± SEMs.

## Additional Information

**How to cite this article**: Yang, J. *et al.* Anomalous uptake and circulatory characteristics of the plant-based small RNA MIR2911. *Sci. Rep.* **6**, 26834; doi: 10.1038/srep26834 (2016).

## Supplementary Material

Supplementary Information

## Figures and Tables

**Table 1:** The quantified amount of miRNA2911 present in five fresh vegetables by qRT-PCR.

Vegetables	MIR2911 (fmoles/g)
Broccoli	301.6
Cabbage	380.0
Cauliflower	548.8
Lettuce	84.3
Spinach	228.3

MIR2911 was measured from fresh vegetables by qRT-PCR.
