# Supplementary material for: Anomalous uptake and circulatory characteristics of the plant-based small RNA MIR2911
Source: Sci Rep. 2016 Jun 2;6:26834. doi: 10.1038/srep26834 (PMC4890004; doi:10.1038/srep26834)
Supplement: Supplementary Information [file srep26834-s1.pdf]

Anomalous uptake and circulatory characteristics of the plant-based small RNA MIR2911

Jian Yang[1], Tremearne Hotz[2], LaCassidy Broadnax[1], Mark Yarmarkovich[3], Ismail Elbaz[1], Kendal D Hirschi[1,4,*]

[1]USDA/ARS Children's Nutrition Research Center, Baylor College of Medicine, 1100 Bates Street, Houston, TX 77030

[2]Bates College, 2 Andrews Rd, Lewiston, ME 04240

[3]Cancer Biology, University of Pennsylvania, 3501 Civic Center Boulevard, Philadelphia, PA 19104

[4]Vegetable and Fruit Improvement Center, Texas A&M University, College Station, TX 77845

*Author to whom correspondence should be addressed: 1100 Bates St, CNRC Room 9016, Houston, Texas 77030; E-mail: kendalh@bcm.edu; Tel.: 713-798-7011; Fax: 713-798-7171

Figure S1

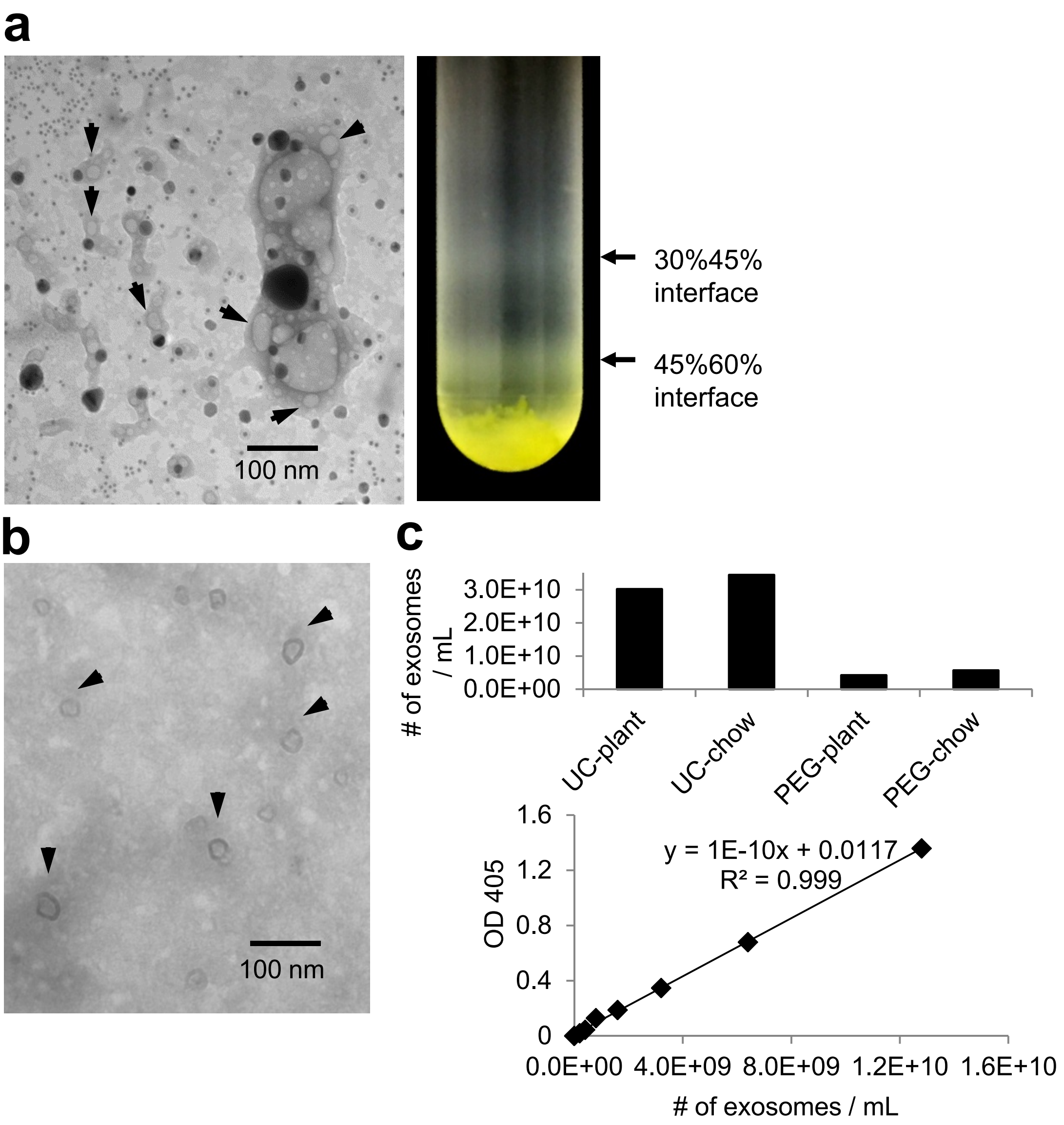

Supplementary information, Figure S1. Characterization of EPDENs and exosomes. **(a)** Left: EPDENs from cabbage. Arrow heads point to the EPDENs. Right: EPDEN bands from sucrose gradient.
(b) Mouse exosomes isolated by ultracentrifugation from mouse serum. Arrow heads point to the exosomes **(c)** Top: Exosomes isolated by Ultracentrifugation protocol (UC) or PEG precipitation protocol (PEG), from mice fed plant based diet (-plant) or chow diet (-chow) were quantified using EXOCRET assay. Bottom: standard curve of serial dilutions of exosomes assayed by EXOCRET assay.

Figure S2

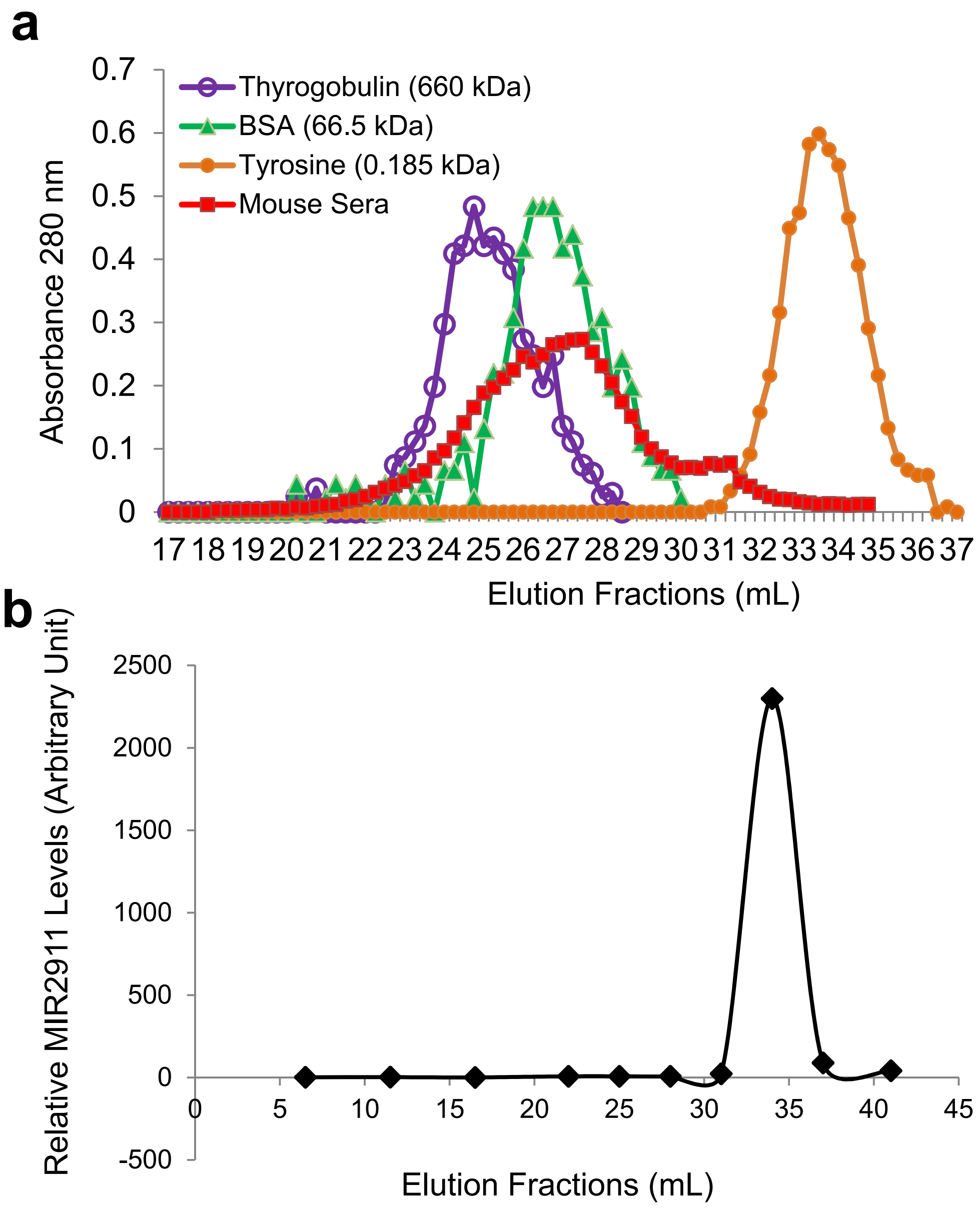

Supplementary information, Figure S2. Size-exclusion chromatography of size standards and control samples. **(a)** Size standards thyroglobulin (660 kDa), Bovine Serum Albumin (66.5 kDa), Tyrosine (0.185 kDa), and human sera were fractionated on a Sephacryl S-500 column. Fractions were assayed for protein concentration by absorbance at 280 nm. **(b)** Synthetic MIR2911 (400 pmoles) were fractionated on the same Sephacryl S-500 column. Levels of MIR2911 in fractions were determined by qRT-PCR.
